# News from Societies

**Published:** 1960-10

**Authors:** 


### *News from Societies*

### PROGRAMMES FOR 1960–61 SESSION

### UNIVERSITY OF BRISTOL

POSTGRADUATE COURSES

The following course is being arranged:

Sunday morning Lecture-demonstrations, from 24th October to 11th December, 1960. Details may be obtained from the Department of Postgraduate Medical Studies.

### BATH CLINICAL SOCIETY

*Secretary:* Dr. J. R. Bolton, 61 Englishcombe Lane, Bath

14th Oct.: **Clinical Meeting** at St. Martin's Hospital, Bath.

11th Nov.: Dr. R. Bishton, Dr. D. W. Pugh, Mr. Stuart-Black Kelly: **Symposium on Diabetes Mellitus**

9th Dec.: C. P. Sames, Esq., M.S., F.R.C.S., **"Peripatetic Proctology"**

13th Jan.: Dr. J. M. Drew, **"From Bed to Worse"**; Dr. J. Beviss, **"From Dizzy Heights"**

10th Feb.: **Clinical Meeting**

10th Mar.: Dr. Richard Asher, M.D., F.R.C.P., **"Hypnosis in General Medicine"**

14th Apr.: Bryce Blair, Esq., F.R.C.S., F.R.O.G., **"Variations on a Theme"**

12th May: **Annual General Meeting**

All Meetings will be held at the Royal United Hospital, Bath, unless otherwise stated.

### B.M.A. (CORNWALL DIVISION)

*Hon. Secretary:* Dr. Eric Townsend, Fieldways, Tregenna Lane, Camborne

24th Nov.: **Annual Dinner and Dance,** Hotel Bristol, Newquay.

24th Mar.: Professor M. L. Rosenheim, **B.M.A. Clinical Lecture,** Royal Infirmary, Truro, 8.30 p.m.

### B.M.A. (GLOUCESTERSHIRE BRANCH)

*Hon. Secretary:* Dr. H. G. Scott-Kerr, 29 Leckhampton Road, Cheltenham

13th Oct.: Mr. J. D. J. Freeman. **Presidential Address: "A Naval Opthalmologist in Ceylon",** and a film, colour and sound, **"Land of the Buddha"** Gloucester.

10th Nov.: **Representatives' Report.** Financial Statement. Film. Cheltenham.

8th Dec.: Guest Speaker: Mr Brian Inglis, Editor of *The Spectator*. Combined Meeting with the Press. Gloucester.

12th Jan.: Dr. P. G. Mann. Director, Public Health Laboratory, Bath. Paper: **"Chemotherapy of Acute Infections"** Cheltenham.

9th Feb.: Dr. D. Lindsay Walker, **"Some Psychiatric Concepts where Emphasis is misplaced".** Gloucester.

9th Mar.: **Clinical Meeting.** Cheltenham.

13th Apr.: P. D. Trevor-Roper, Esq., M.D., D.O.M.S., F.R.C.S. (Doctors' Ladies to be invited). B.M.A. Lecturer. Address: **"Influence of Eye Disease on the Artist",** illustrated with slides. Gloucester.

11th May: Mr. R. J. Hart. **Annual Meeting and Paper: "Problems in the Early Diagnosis of Bone Disease".**

8th June: Dr. Richard Sydney Allison, Senior Physician, Royal Victoria Hospital, Belfast. **President's Guest.** Gloucester.

Unless otherwise stated the meetings will be held at 6.15 p.m. The place of the meetings will be stated in the circular notices.

### B.M.A. (SOUTH-WESTERN BRANCH)

*Hon. Secretary:* Dr. F. E. Graham-Bonnalie, 37 The Strand, Topsham, Nr. Exeter

17th Nov.: **Combined Meeting** with the Exeter and South Devon Medico-Chirurgical Society.

3rd Dec.: **Autumn Clinical Meeting** at Plymouth.

### BRISTOL NORTH AND WEST GROUP OF GENERAL MEDICAL PRACTITIONERS

*Hon. Sec.:* Dr. C. Jean Fraser, 20 Dragon's Well Road, Henbury, Bristol

Meetings on the second Tuesday of each month during winter. Programme to be arranged.

### PLYMOUTH MEDICAL SOCIETY

Meetings at 8.30 p.m.

*Secretaries:* Dr. Bernard Peck, 5 Lockington Avenue, Hartley, Plymouth and Mr. Michael Reilly, Cresswell, Hartley Avenue, Plymouth.

14th Oct.: Dr. J. W. Landells, M.R.C.P., **"The Dangers of Medical Treatment"**, North Friary House.

28th Oct.: **Clinical Evening** (Cases from 8 p.m.), Freedom Fields Hospital.

4th Nov.: G. L. Alexander, Esq., F.R.C.S., **"The Danger Signs in Cases of Head Injury"**, North Friary House.

18th Nov.: Dr. Richard Asher, M.D., F.R.C.P., **"See How They Come"**, Joint Meeting with Plymouth Division, B.M.A., North Friary House.

23rd Nov.: **Annual Dinner and Dance,** Duke of Cornwall Hotel.

9th Dec.: **"Mutual Problems"**, Joint Discussion Meeting with Plymouth Section, British Dental Association, North Friary House.

20th Jan.: Prof. J. Chassar Moir, D.M., F.R.C.S., F.R.C.O.G., **"Some English Queens and their Confinements"**, North Friary House.

3rd Feb.: Prof. J. H. Burn, M.D., F.R.S., **"The Action of Reserpine in Peripheral Vascular Disease"**, North Friary House.

14th Feb.: **Clinical Evening.** (Cases from 8 p.m.), Royal Naval Hospital.

3rd Mar.: **Short Papers** by: Dr. G. B. Carter, Dr. T. L. Chester Williams, Mr. I. Sutherland-Jones and Dr. B. J. Peck, North Friary House.

4th Apr.: **Clinical Evening** (Cases from 8 p.m.), Freedom Fields Hospital.

3rd May: Visit to Ellbridge Experimental Horticultural Station. (Members' Ladies also invited), Nr. Saltash.

8th May: **Annual General Meeting,** North Friary House.

### COSSHAM MEDICAL SOCIETY

*Secretary:* Dr. K. W. Moulding, 235 Lodge Causeway, Bristol

4th Oct.: Mrs. Z. M. U. Eastes, M.R.C.S., L.R.C.P., **Presidential Address.**

1st Nov.: H. Leather, Esq., M.D., M.R.C.P., **"Medicine in Africa".**

6th Dec.: H. L. Shepherd, Esq., M.B., CH.M., F.R.O.G., **"Maternal Mortality".**

17th Dec.: Cheese and Wine Party at Marshfield.

3rd Jan.: S. F. Marwood, Esq., M.D., M.R.C.P., **"Treatment in Diabetes—a review".**

7th Feb.: A. C. Hunt, Esq., M.D., B.S., **"Unnatural Deaths".**

7th Mar.: Miss B. D. Corner, M.D., F.R.C.P., **"Early Arrivals, what are the later results?".**

4th Apr.: **Annual General Meeting.**

### ROYAL DEVON AND EXETER HOSPITAL POSTGRADUATE PROGRAMME

*Secretary:* Mr. H. Dendy Moore, 4 Magdalen Road, Exeter

The Exeter Postgraduate Meetings will take place on Tuesdays at 8.30 p.m. from 11th October to 6th December, 1960.

### SOCIETY OF MEDICAL OFFICERS OF HEALTH WEST OF ENGLAND BRANCH

*Secretary:* Dr. R. H. G. H. Denham, Council Offices, 8 Cleveland Place, East, Bath.

The Society is divided into two sections, Northern and Southern. Each section holds four meetings a year, one of which being a joint meeting of both sections.

1st Oct.: E. D. Irvine, Esq., M.D., D.P.H., M.O.H., Exeter. **Presidential Address.** (Taunton).

7th Jan.: At Bristol. (details later).

1st Apr.: **Joint Meeting** of Northern and Southern Sections. (Details later).

June. At Bath. (Details later).

### WESSEX RAHERE CLUB

The autumn dinner of the above club will take place on 29th October, 1960, at the Grand Spa Hotel, Clifton, Bristol, under the Chairmanship of Dr. Russell Grant of Winchester. It is hoped the Guest of Honour will be Dr. Heber Langston. Further details will be circulated or can be obtained by any Barts. graduates who are not already members from the Hon. Secretary, Mr. A. Daunt Bateman, F.R.C.S., 11 The Circus, Bath.

